# Physio-biochemical and ultrastructural impact of (Fe_3_O_4_) nanoparticles on tobacco

**DOI:** 10.1186/s12870-019-1864-1

**Published:** 2019-06-13

**Authors:** Rami Alkhatib, Batool Alkhatib, Nour Abdo, Laith AL-Eitan, Rebecca Creamer

**Affiliations:** 1 Department of Biotechnology and Genetic Engineering, Faculty of Science and Arts, Jordan University of Science and Technology, Irbid, 22110 Jordan; 2 Molecular Biology Program, New Mexico State University, Las Cruces, NM 88003 USA; 3 Department of Public Health, Faculty of Medicine, Jordan University of Science and Technology, Irbid, 22110 Jordan; 4 Department of Entomology, Plant Pathology and Weed Science, New Mexico State University, Las Cruces, NM 88003 USA

**Keywords:** Chloropyll content, Iron oxide, Leaf area, Photosynthetic rate, Transpiration rate, Transmission microscope, Xylem

## Abstract

**Background:**

Because of their broad applications in our life, nanoparticles are expected to be present in the environment raising many concerns about their possible adverse effects on the ecosystem of plants. The aim of this study was to examine the effect of different sizes and concentrations of iron oxide nanoparticles [(Fe_3_O_4_) NPs] on morphological, physiological, biochemical, and ultrastructural parameters in tobacco (*Nicotiana tabacum* var.2 Turkish).

**Results:**

Lengths of shoots and roots of 5 nm-treated plants were significantly decreased in all nanoparticle-treated plants compared to control plants or plants treated with any concentration of 10 or 20 nm nanoparticles. The photosynthetic rate and leaf area were drastically reduced in 5 nm (Fe_3_O_4_) NP-treated plants of all concentrations compared to control plants and plants treated with 10 or 20 nm (Fe_3_O_4_) NPs. Accumulation of sugars in leaves showed no significant differences between the control plants and plants treated with iron oxide of all sizes and concentrations. In contrast, protein accumulation in plants treated with 5 nm iron oxide dramatically increased compared to control plants. Moreover, light and transmission electron micrographs of roots and leaves revealed that roots and chloroplasts of 5 nm (Fe_3_O_4_) NPs-treated plants of all concentrations were drastically affected.

**Conclusions:**

The size and concentration of nanoparticles are key factors affecting plant growth and development. The results of this study demonstrated that the toxicity of (Fe_3_O_4_) NPs was clearly influenced by size and concentration. Further investigations are needed to elucidate more about NP toxicity in plants, especially at the molecular level.

**Electronic supplementary material:**

The online version of this article (10.1186/s12870-019-1864-1) contains supplementary material, which is available to authorized users.

## Background

Nanoparticles are widely used in many industrial, commercial, environment, agriculture, and biomedical sectors [1, 2]. Their utility is based on their constant physical properties, which is strictly dependent on their size, which varies from 1 to 100 nm [3, 4]. The effects of nanoparticles on living systems, especially, on plants, have been the focus of many studies, however, little is known about the specific impacts of nanoparticles on specific plant systems. The impact of nanoparticles on different plant species can vary greatly, and both positive and negative effects have been reported. Although plants are primary producers and play major roles in the ecosystem, the impact of nanoparticles upon them is not well studied [5].

Nanoparticles (NPs) interact with plants causing several morphological, physiological, and biochemical changes, which depends on properties of nanoparticles, including concentration, size, and their physical properties, as well as the plant species tested [6]. Pumpkin plants were shown to take up (Fe_3_O_4_) NPs which accumulated in various tissues [7]. Moreover, iron oxide NPs affect chlorophyll content and the efficiency of the photosynthetic apparatus in soybean plants and might have influence on both biochemical and enzymatic reactions [8]. In corn (*Zea mays* L.), γ-Fe_2_O_3_ NPs (particle size 17.7 ± 3.9 nm) at 20 mg/L under hydroponic conditions significantly promoted root elongation, while 50 and 100 mg/ L γ-Fe_2_O_3_ NPs significantly decreased root length, indicating that the impact of NPs on plants depends on their concentration [9]. In contrast, sunflower (*Helianthus annuus* L.) treated with γ-Fe_2_O_3_ NPs (particle size 20–100 nm) at 50 and 100 mg/L under hydroponic conditions had decreased concentrations of the macronutrients Ca, K, Mg, and S in the shoot, decreased chlorophyll pigments, and reduced root function [10].

The aim of this study was to investigate the impact of applications of different sizes and concentrations of (Fe_3_O_4_) NPs on the morphological, physiological, biochemical, structural and ultrastructural characteristics of tobacco.

## Results

**Fig. 1:**
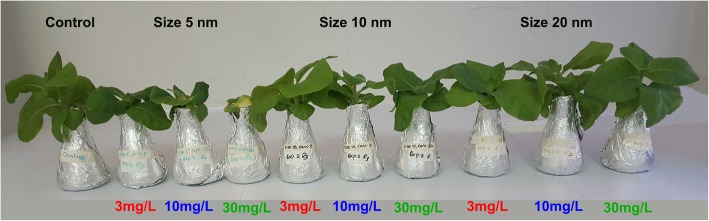
Effect of different sizes and concentrations of (Fe_3_O_4_) NPs in the growth and development of *Nicotiana tabacum* shoots as compared to control plants. Control, size 5 nm (Fe_3_O_4_) [3, 10, 30 mg/L concentrations]; size 10 nm (Fe_3_O_4_) [3, 10, 30 mg/L concentrations]; and size 20 nm (Fe_3_O_4_) [3, 10, 30 mg/L concentrations]

**Fig. 2:**
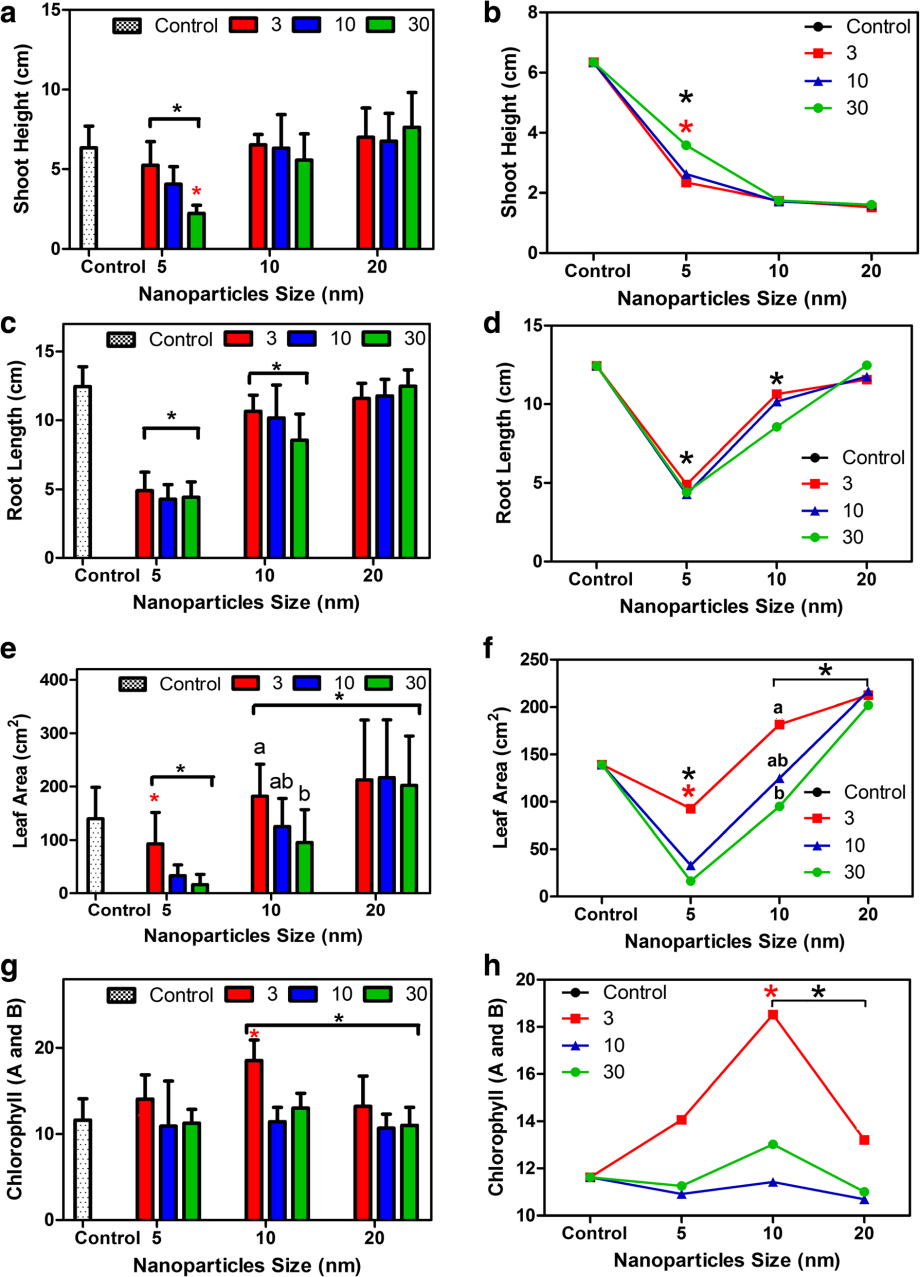
Changes in the morphological and physiological parameters and the trend of each parameter in response to size and concentration of (Fe_3_O_4_) NPs-treated plants as compared to control plants. Shoot height (cm) (**a** and **b**); Root length (cm) (**c** and **d**); Leaf area (cm^2^) (**e** and **f**); Chlorophyll content (**g** and **h**). (*P* < 0.0001 for all parameters) in response to (Fe_3_O_4_) NPs and their concentrations. * indicates values that are significantly different from the control (Post-hoc tukey test with *P* < 0.05)

The overall physical appearance of shoots from plants treated with 10 and 20 nm NPs, in all concentrations, were similar to those of the control plants (Fig. 1). In contrast, shoot height and root length of 5 nm NPs-treated plants were significantly decreased (*p <* 0.0001) (Fig. 1 and Fig. 2) compared to all treated plants and control plants. Moreover, plants treated with 10 nm iron oxide NPs also showed a significant reduction in root length compared to control plants and 20 nm iron oxide-treated plants (Fig. 2c and d; Additional file 1: Figure S1)). The leaf area of plants treated with 5 nm iron oxide was significantly reduced compared to all other treated and control plants and also showed some leaf chlorosis. However, the leaf area increased in plants treated with 20 nm iron oxide (Fig. 2e and f). For Chl a + b, there was no significant differences between control plants and iron oxide-treated plants. However, plants treated with 10 nm iron oxide particles had significantly higher Chl a + b than those treated with 20 nm iron oxide. In the plants treated with size 10 nm iron oxide, the Chl a + b content in plants treated with the 3 mg/L concentration was the highest (Fig. 2g and h).

**Fig. 3:**
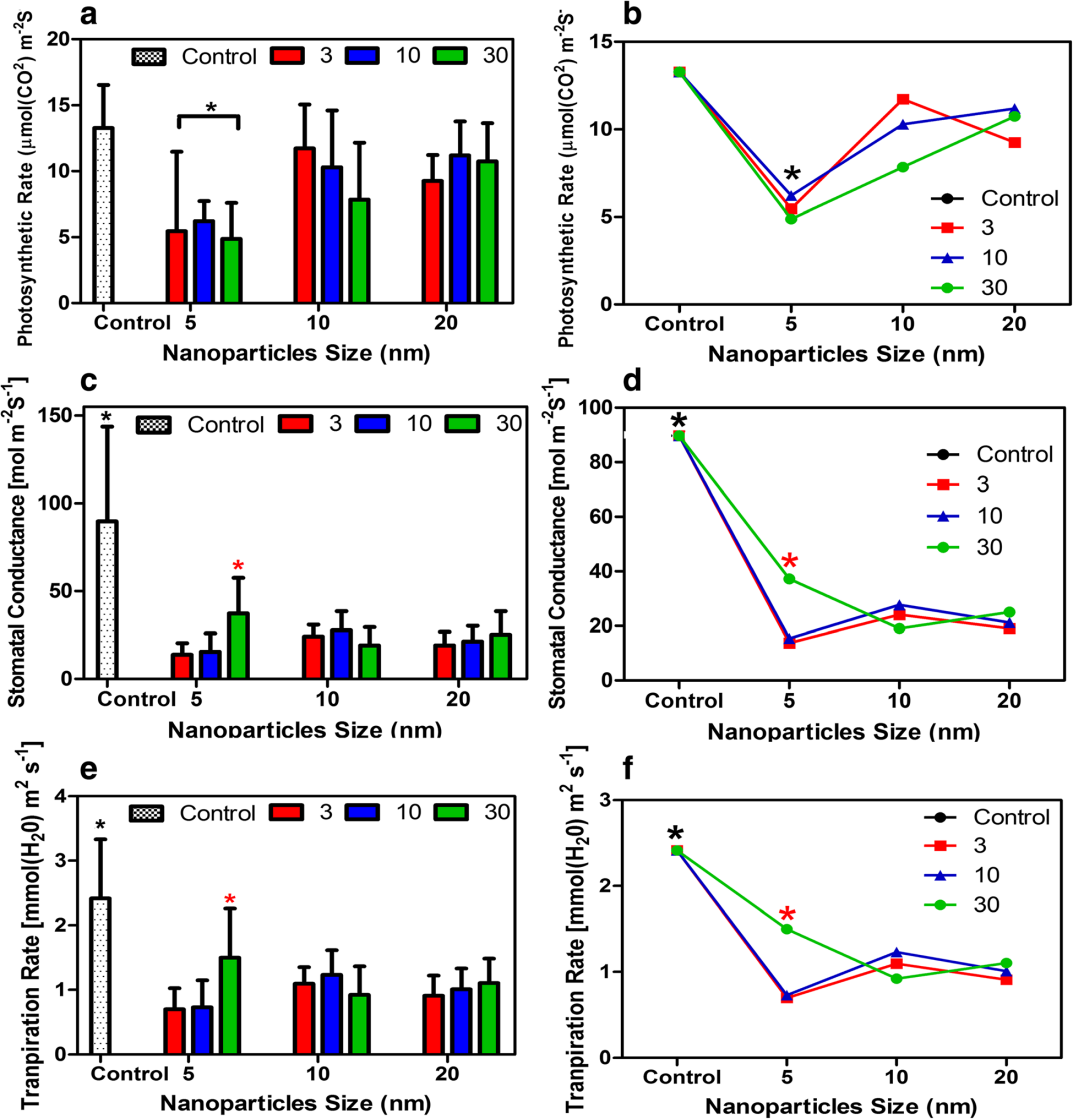
Changes in photosynthetic parameters in *Nicotiana tabacum* and the trend of each parameter in response to size and concentration of (Fe_3_O_4_) NPs-treated plants as compared to control plants. Photosynthetic rate [μmol m^− 2^ s^− 1^] (**a** and **b**). Stomatal conductance [mol (H_2_O) m^− 2^ s^− 1^] (**c** and **d**). Transpiration rate [mmol(H_2_O) m^− 2^ s^− 1^] (**e** and **f**) . Data represent the means (± SE) of three independent experiments. * indicates values that are significantly different from the control (Post-hoc tukey test with *P* < 0.05)

**Fig. 4:**
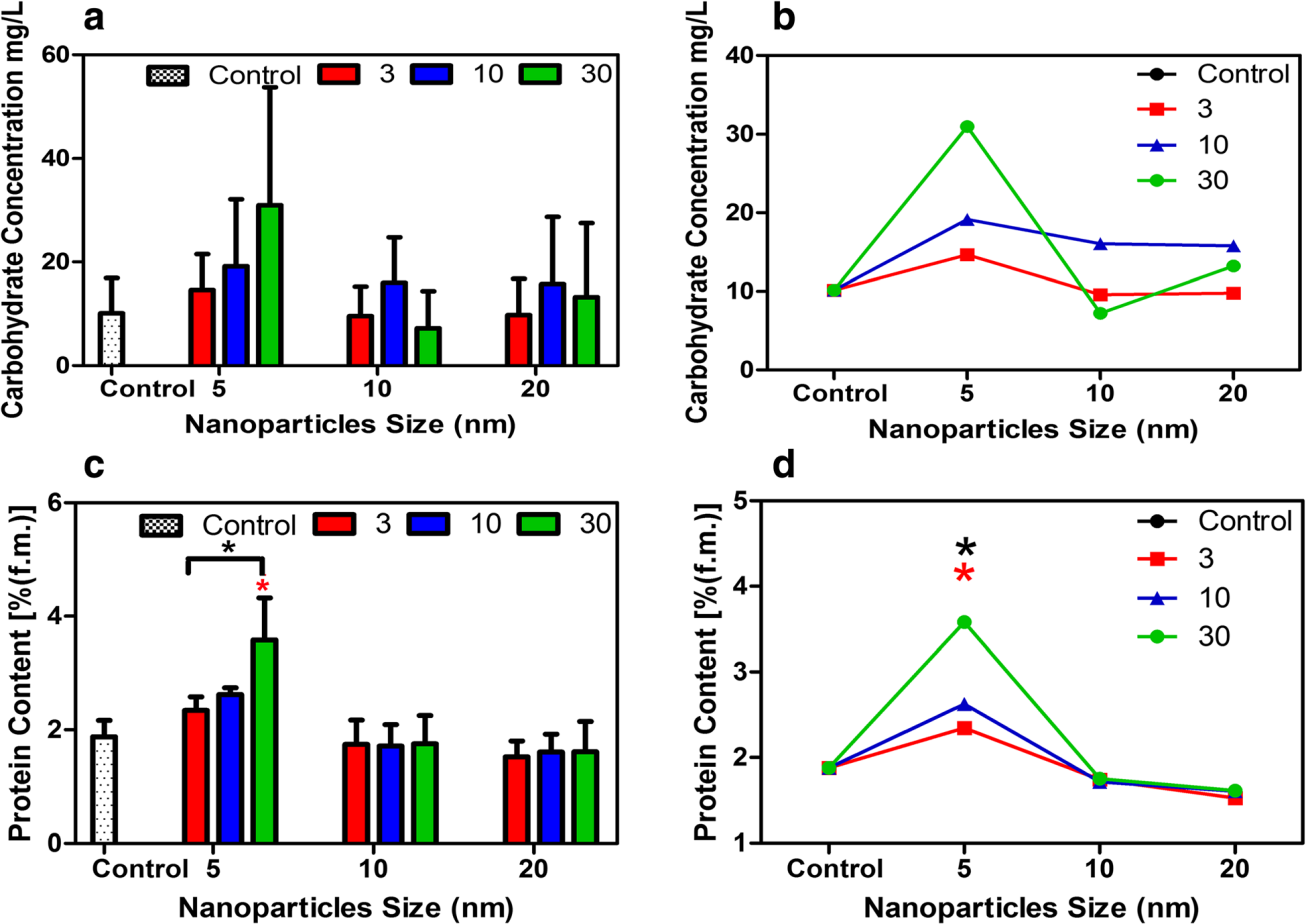
Changes in biochemical parameters and the trend of each parameter in response to size and concentration of (Fe_3_O_4_) NPs-treated plants as compared to control plants . Sugar content [mg/L] (**a** and **b**). Protein content [% (f.m)] (**c** and **d**) as compared to control plants. (*P* < 0.0001 for all parameters) in response to (Fe_3_O_4_) NPs and their concentrations. * indicates values that are significantly different from the control (Post-hoc tukey test with *P* < 0.05)

Further analyses were performed by Infrared gas analyzer (IRGA) portable photosynthetic system. The photosynthetic rate (*P*_N_) for control plants was 13.3 [μmol m^− 2^ s^− 1^]. In contrast, *P*_N_ significantly decreased in 5 nm-treated plants in all concentrations (5.5, 6.2, and 4.8, respectively [μmol m^− 2^ s^− 1^]) (*P* < 0.0001). No significant changes in *P*_N,_ *gs* and *E* were found for leaves in plants treated with 10 and 20 nm iron oxide particles compared to control plants (Fig. 3a and b). Interestingly, both *gs* and *E* exhibited a significant drop in leaves grown in 5 nm (Fe_3_O_4_) nanoparticles at 30 mg/L concentration at day 14 as compared with control plants (*P* < 0.0001) (Fig. 3c, d, e, f). Accumulation of sugars in leaves was not significantly different among the control and plants treated with iron oxide in all sizes and concentrations (Fig. 4a and b). However, plants treated with 5 nm NP (30 mg/L concentration) exhibited the highest carbohydrate content compared to control and all treated plants (all NP sizes and concentrations). Protein accumulation in plants treated with 5 nm iron oxide dramatically increased, especially in the 30 mg/L treated plants compared to control plants (*P* < 0.001) (Fig. 4c and d).

**Fig. 5:**
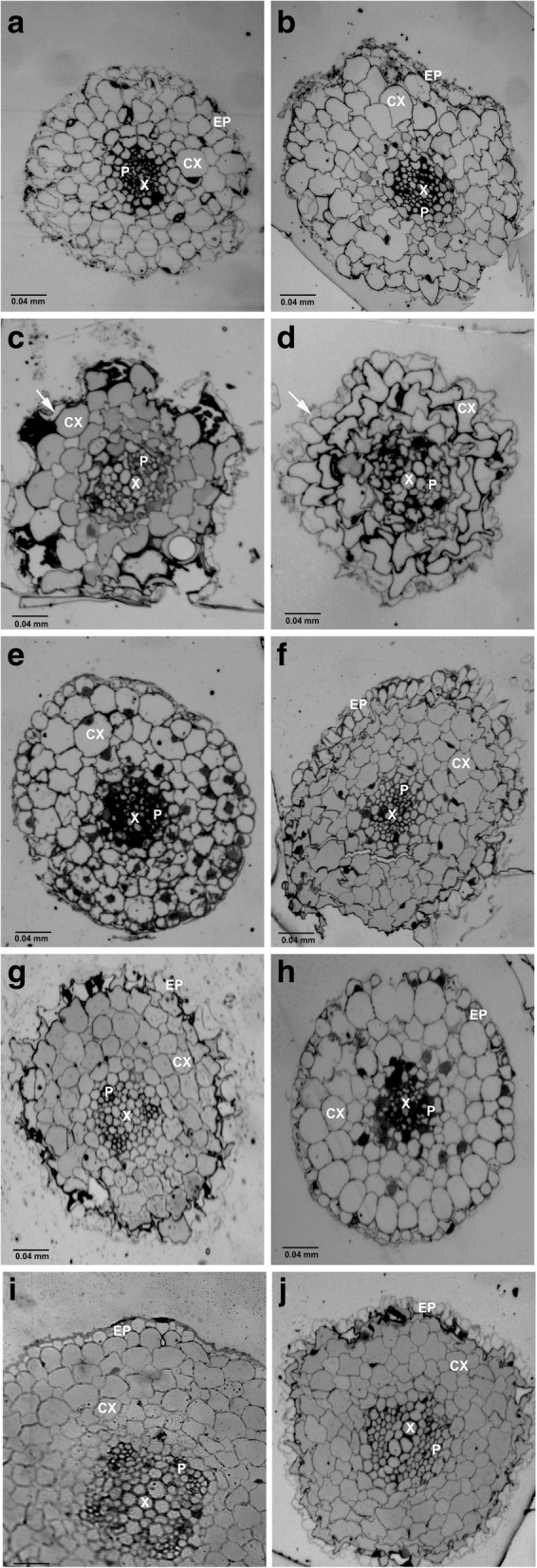
Light microscopic images showing thick sections of *Nicotiana tabacum* root. Control root (**a**). 5 nm (Fe_3_O_4_) NPs-treated plants with 3, 10, and 30 mg/L (**b**, **c**, and **d**, respectively). 10 nm (Fe_3_O_4_) NPs treated plants with 3, 10, and 30 mg/L (**e**, **f**, and **g**, respectively). 20 nm (Fe_3_O_4_) NPs treated plants with 3, 10, and 30 mg/L (**h**, **i**, and **j**, respectively). 5 nm (Fe_3_O_4_) NPs treated plants showing deformation of the epidermis (EP) *(arrow)*; cortical cells (CX) and thickened xylem (X) and showing major disruption in the organization of the cortical cells (CX) and vascular bundles [xylem (X) and phloem (P)]. Bar equals 0.04 mm

Light microscopy of roots for control plants (Fig. 5a) exhibited normal, regular-shaped epidermal cells with multi-layer cortical cells. In addition, well-arranged xylem and phloem cells were found inside the vascular tissue. In contrast, 5 nm iron oxide-treated plants at concentrations 10 and 30 mg/ml showed deformed epidermal and cortical cells with reduced number of cortical cell layers (Fig. 5c and d) compared with the control root thick sections. Moreover, plants treated with 10 and 20 nm iron oxide in all concentrations showed no visible deformation in their structural characteristics (Fig. 5e, f, g, h, i).

**Fig. 6:**
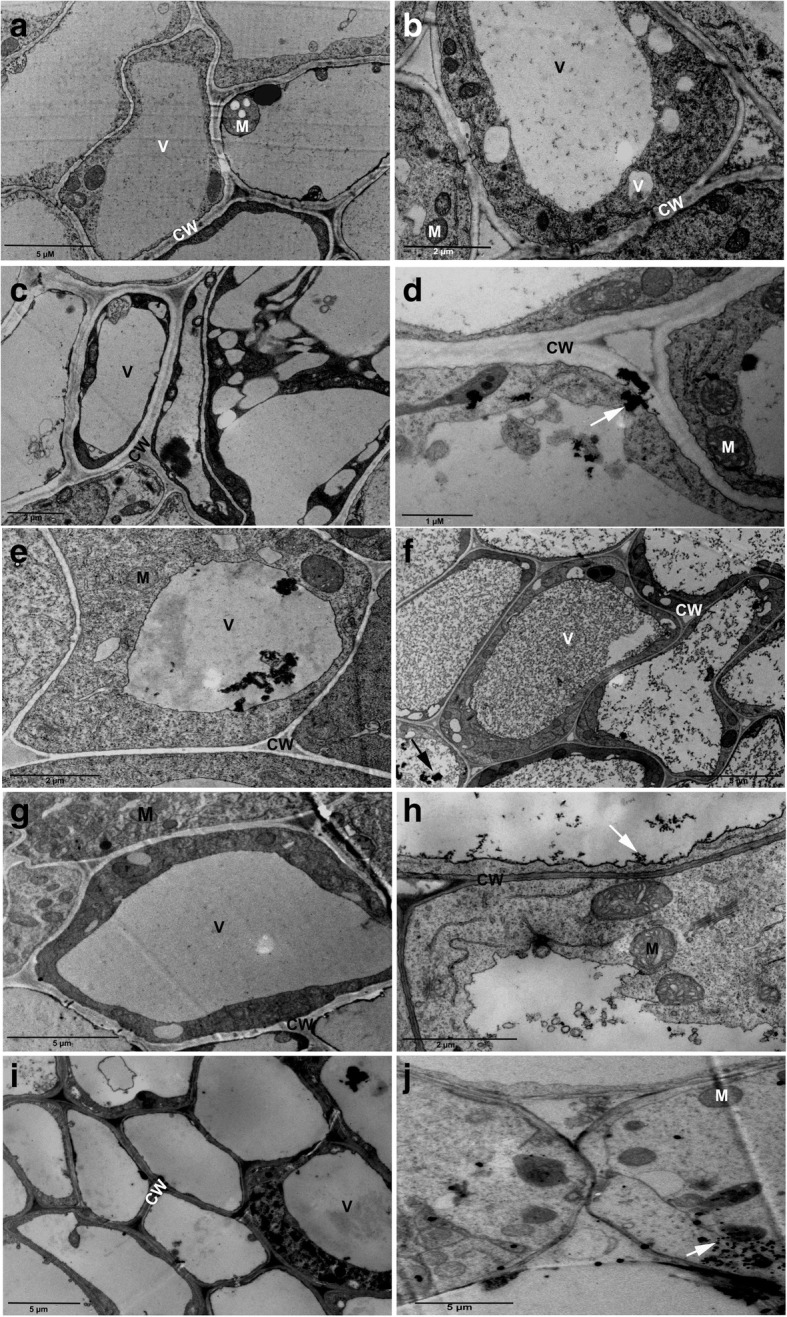
Transmission electron micrographs of *Nicotiana tabacum* root cortical cells of control and (Fe_3_O_4_) NPs -treated plants. Control plant (**a)**. 5 nm (Fe_3_O_4_) NPs-treated plants with 3, 10, and 30 mg/L (**b**, **c**, and **d**, respectively). 10 nm (Fe_3_O_4_) NPs treated plants with 3, 10, and 30 mg/L (**e**, **f**, and **g**, respectively). 20 nm (Fe_3_O_4_) NPs-treated plants with 3, 10, and 30 mg/L (**h**, **i**, and **j**, respectively). NPs (Arrow), Mitochondria (M), cell wall (CW), small vacuoles (V)

TEM-micrographs of control plant root system showed normal vacuole and mitochondria with normal cristae (Fig. 6a). In contrast, the TEM-micrographs of the root system grown under 5 nm iron oxide (3 and 10 mg/L) exhibited some vacuolation (Fig. 6b and c). Moreover, unstained TEM-micrographs in 5 nm NPs-treated plants at 30 mg/L concentration showed mitochondria with stretched cristae system near the plasma membrane and clustering of iron oxide NPs in the cell wall (Fig. 6d). Plants treated with 10 nm iron oxide (3, 10, and 30 mg/L) exhibited normal mitochondria. Iron oxide nanoparticles were visible inside the vacuole and near the plasma membrane (Fig. 6e, f, g). Plants treated with 20 nm iron oxide in all concentrations showed normal ultrastructural characteristics with visible NP affinity to the plasma membrane of the root system (Fig. 6h, i, f).

**Fig. 7:**
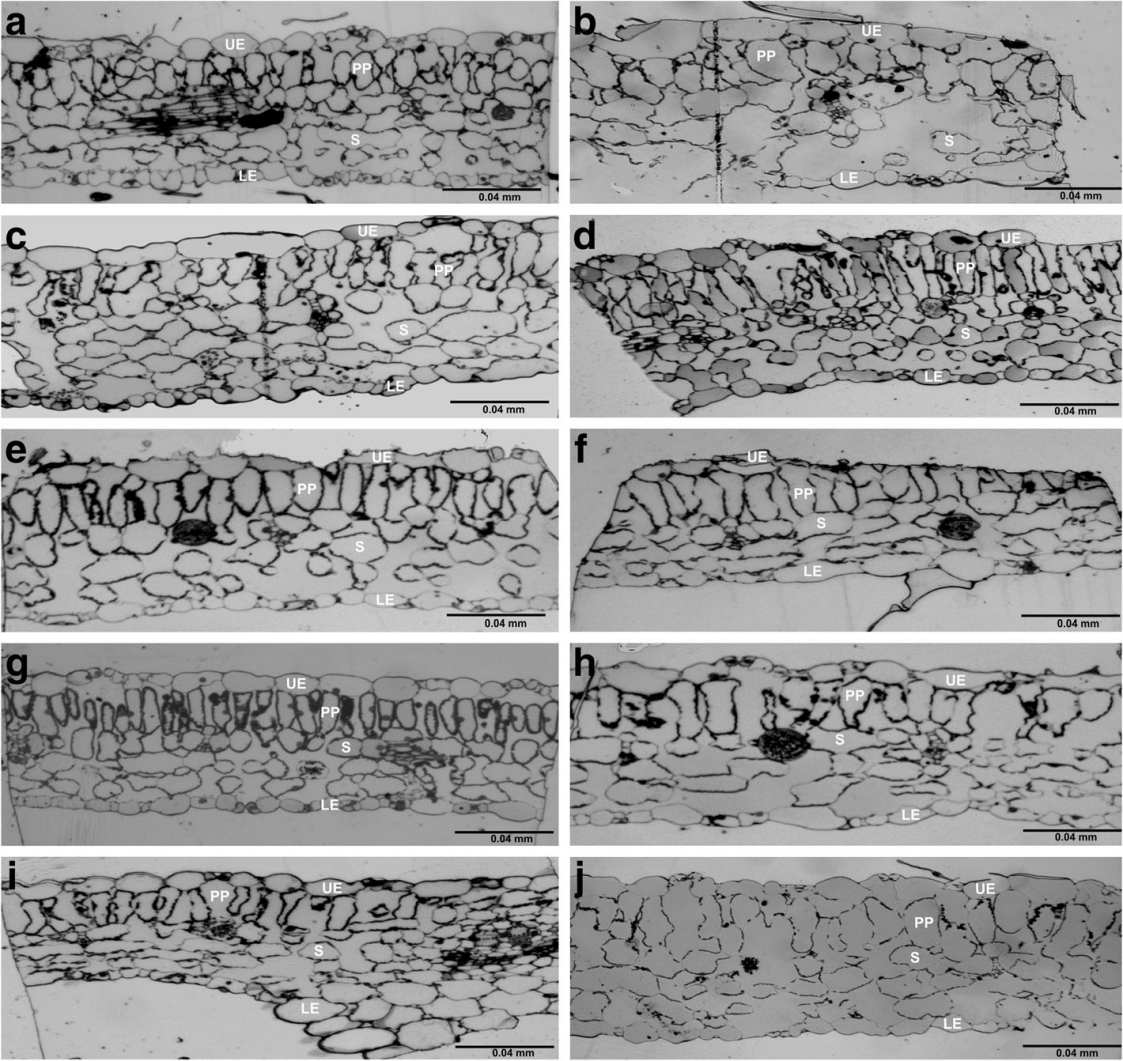
Thick leaf sections of *Nicotiana tabacum* control and (Fe_3_O_4_) NPs -treated plants. Micrograph images of control plant (**a**). 5 nm (Fe_3_O_4_) NPs-treated plants with 3, 10, and 30 mg/L (**b**, **c**, and **d**, respectively). 10 nm (Fe_3_O_4_) NPs-treated plants with 3, 10, and 30 mg/L (**e**, **f**, and **g**, respectively). 20 nm (Fe_3_O_4_) NPs-treated plants with 3, 10, and 30 mg/L (**h**, **i**, and **j**, respectively). Upper epidermis (UE), palisade parenchyma layer (PP), spongy parenchyma layer (S), lower epidermis (LE). Bar equals 0.04 mm

**Fig. 8:**
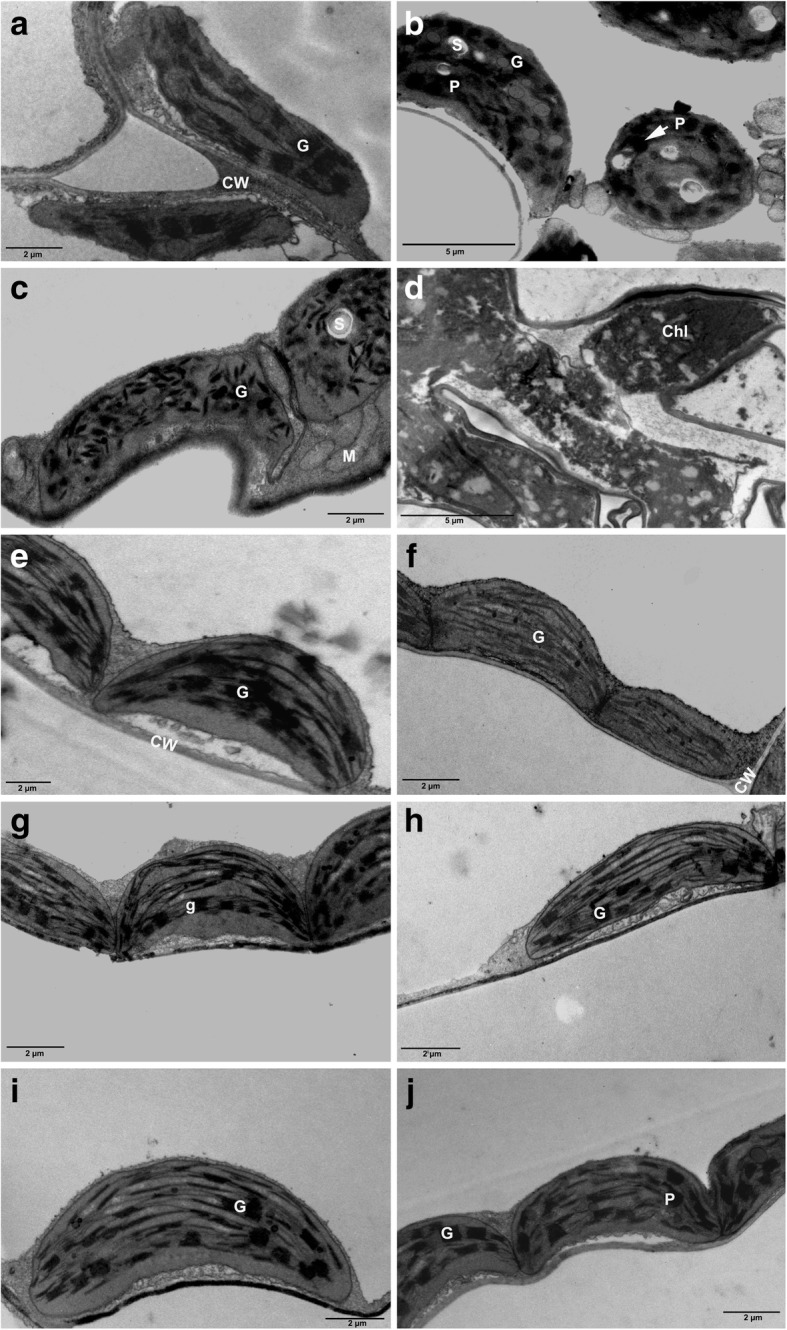
Thin leaf sections of *Nicotiana tabacum* control and (Fe_3_O_4_) NPs-treated plants. Micrograph images of control plant (**a**). 5 nm (Fe_3_O_4_) NPs-treated plants with 3, 10, and 30 mg/L (**b**, **c**, and **d**, respectively) exhibited deformed thylakoids membrane. 10 nm (Fe_3_O_4_) NPs-treated plants with 3, 10, and 30 mg/L (**e**, **f**, and **g**, respectively). 20 nm (Fe_3_O_4_) NPs-treated plants with 3, 10, and 30 mg/L (**h**, **i**, and **j**, respectively). Cell wall (CW), chloroplast (Chl), grana (G), mitochondria (M), Plastoglobule (P)

The light microscopy images of control leaves and 5 nm iron oxide treated-leaves (3, and 10 mg/L) showed normal, regular-shaped epidermal cell layers, and well-organized palisade and spongy layers (Fig. 7a, b, c). However, 5 nm-treated leaves (30 mg/L) showed distorted epidermal cells, and palisade and spongy layers (Fig. 7d). In contrast, leaves exposed to 10 and 20 nm iron oxide in all concentrations exhibited well-organized palisade and spongy layers (Fig. 7e, f, g, i, h). Similarly, TEM-micrographs of the leaves of control plants and those treated with 10 and 20 nm iron oxide in all concentrations, revealed no differences in their chloroplasts (Fig. 8a, e, f, g, h, i, f). In contrast, the integrity of thylakoid membranes of chloroplasts in plants treated with 5 nm iron oxide (3, 10 mg/L) was lost compared to control plants (Fig. 8b, and c), while plants treated with 5 nm (30 mg/L) were extremely reduced, and showed loss of integrity of thylakoid membranes (Fig. 8d).

## Discussion

The toxicity of nanomaterials and their negative effects on growth and development of plants have not been fully elucidated. Hormesis, in toxicology, is defined as a biphasic response to a toxic compound (stressor), which at low doses induces a beneficial effect (eustress) and at high doses it is deleterious. However, the mechanism of hormesis in plants is still unknown [11]. Our data showed that 5 nm-(Fe_3_O_4_) NPs treated shoots and roots at any concentration exhibit reductions in shoot height and root length, and these reductions increased as NP concentration increased. This suggests an excess absorption of these particles compared to other sizes used in this study which may explain the toxic effects shown, including the decrease in growth and irregularities in cell division [12–14]. Severe chlorosis on leaves of plants treated with 30 mg/L of 5 nm iron oxide was observed, which suggests that total chlorophyll contents were affected although not significantly compared to control plants. In contrast, total chlorophyll content in plants treated with 10 and 20 nm (Fe_3_O_4_) NPs, in all concentrations, was significantly increased compared to control and plants treated with 5 nm (Fe_3_O_4_) nanoparticles in all concentrations. Higher ethylene causes an increase in activity of the chlorophyllase enzyme and destruction of internal chloroplast membranes [15], which may explain the higher contents of chlorophyll in plants treated by 10 and 20 nm (Fe_3_O_4_) NPs if the treatment inhibited ethylene.

In this study, the photosynthetic parameters examined were net photosynthetic rate, stomatal conductance, and transpiration rate. The net photosynthetic rate (*P*_N_) was significantly reduced in 5 nm (Fe_3_O_4_) NP-treated plants in all concentrations. However, no significant drop in (*P*_N_) was observed in plants treated with 10 and 20 (Fe_3_O_4_) NP compared to control plants. Moreover, *g*_*s*_ and *E* in plants treated with 5 nm (Fe_3_O_4_) nanoparticles were not affected compared to control plants and 10 and 20 nm (Fe_3_O_4_) NP-treated plants in all concentrations. This suggests that *g*_*s*_ and *E* might not be the main regulating factors in seedling retardation and that the influence of (Fe_3_O_4_) NPs on the photosynthetic apparatus is indirect, even though in plants treated with 30 mg/L of 5 nm (Fe_3_O_4_) NPs, *P*_N_ was correlated with the drop in *g*_*s*_ and *E*. This suggests a shift of Rubisco activity towards oxygenation reactions rather than carboxylation [16]. In addition, our data demonstrated that the drop in the *P*_N_ in plants treated with 5 nm NPs in all concentrations could be related to the significant decrease in leaf area which decreases the total amount of light harvested and electron transport component as compared with control and treated plants. Sugar accumulation in leaves is often correlated with decreased activity of Rubisco [17]. Furthermore, the synthesis of osmolytes in plants undergoing abiotic stress is increased significantly to adapt with the stress. Previously, we demonstrated that a drastic reduction of *P*_*N*_ is accompanied by an increase in sugar content in the leaves of 1000 and 5000 μM caffeine-treated plants [18]. Interestingly, our data showed that no significant change in sugar accumulation occurred in plants treated with (Fe_3_O_4_) NPs compared to control plants. However, plant treated with 5 nm NPs showed an increase in sugar content compared to control and other treatment plants. Treating watermelon plants with different concentrations of γ-Fe_2_O_3_ NPs caused no significant changes in sugar accumulation compared to control plants [19]. In contrast, in this study protein production was significantly higher in plants treated with 5 nm (Fe_3_O_4_) NPs in all concentrations, especialy 30 mg/L as compared to control and plants treated with other sizes of NP. This suggests a mechanism of defense-related proteins to cope with the accelerated production of ROS [20]. Moreover, this could be related to the size of the nanoparticle which is the key factor in determining the type and magnitude of the cellular response.

The pore sizes of plant walls are typically in the range of 3–8 nm [21]. However, the internalization of large NPs through cell walls can be induced, allowing these particles to reach vascular tissues (xylem) and translocating through stems to leaves [22, 23]. Plants treated with 5 nm (Fe_3_O_4_) NPs in all concentrations showed deformed epidermal cells, thickened cell walls in the vascular tissues (mainly xylem), impaired shape of the cells, and reduced number of cortical cell layers. This suggests that plants strongly responded to treatment with 5 nm (Fe_3_O_4_) NPs, which was highly toxic, especially at 30 mg/L concentration. Furthermore, TEM-micrographs revealed that 5 nm (Fe_3_O_4_) NPs-treated plants also showed increased vacuolation in the cortical region of the root. Moreover, plants under salt or water stresses exhibit vacuolation in the root cells which might be beneficial for both the accumulation of osmotically active substances and osmotic adjustment [24, 25]. The NPs may leak into nearby tissues and cells (especially cytoplasm and vacuoles) so that little or no translocation occurred [26].

Cross-sectioned leaves of plants treated with 5 nm (Fe_3_O_4_) NPs (30 mg/L concentration) showed some deformation of the spongy parenchyma cells. In contrast, all other treated plants showed no deformation compared to controls. The ultrastructural organization of the leaf chloroplasts in plants treated with 10 and 20 nm (Fe_3_O_4_) NPs was similar to those in control plants. In contrast, deformed chloroplasts of different shapes were observed in plants treated with 5 nm (Fe_3_O_4_) NPs in all concentrations. This suggests that 5 nm (Fe_3_O_4_) NPs were the most effective and toxic among all sizes used in this study, and their toxicity increased as their concentration increased causing growth deformation in plants.

## Conclusions

This study examined the effect of different sizes and concentrations of (Fe_3_O_4_) NPs and their impact on physiological, biochemical, and ultrastructural properties of tobacco. Our findings confirmed that the effect of NPs on tobacco plants depends on size and concentration and suggests that more studies on the effects of NPs on different plant species are needed to elucidate the specific physiological effects of NPs in specific plants.

## Methods

### Plant materials and growth conditions

Tobacco seeds (*Nicotiana tabacum* var. Turkish) were purchased from Altin Tohumculuk Company (Konak, Izmir, Turkey). Two week-old seedlings of 5 cm height and the same number of leaves were carefully removed from the soil and transferred to a hydroponic system-Hoagland’s nutrient solution- (Caisson Laboratories Inc., North Logan, UT, USA), in a growth chamber (Bionex, model VS-3DM, Bucheon, Korea) at a16-h photoperiod, a photosynthetic photon flux density of 250 to 300 μmol m^− 2^ s^− 1^, day/night temperatures of 30/23 °C, and relative humidity of 60/30%. TEM-checked (Fe_3_O_4_) NP of three different sizes (5, 10, and 20 nm) from Sigma-Aldrich (St. Louis, Missouri, USA) were prepared in three different concentrations 3, 10, and 30 mg/L for each size. ImageJ measurements were taken to confirm the internalization of these NPs inside the treated plants (Additional file 1: Figure S1). The experimental design was a randomized complete block, with three treatments and three replicates per treatment.

### Morphological parameters

Shoot heights and root lengths were measured using a metric ruler. Leaf area (cm^2^) was measured for all control and iron oxide-treated plants using a Licor leaf area device (Model 3100 Area Meter, Lincoln, Nebraska, USA).

### Chlorophyll content

Five leaf discs (1.0 cm diameter) were excised from two mature leaves avoiding mid-ribs. Discs were carefully ground with a pestle in 5 ml 80% acetone on ice. Then, extracts were refrigerated in the dark for 2 h. The extracts were transferred to a centrifuge tube. An additional 5 mL of 80% aqueous acetone solution was used to wash the pestle and added to the extract. Extracts were centrifuged for 20 min at high speed (approximately 500x g). The supernatant solution was decanted and the volume brought to 10 mL with 80% aqueous acetone. Spectrophotometric analyses of all samples were determined at 645 nm and 663 nm (UV-M51 UV-VIS, BEL, BEL Engineering, Monza, Italy) and used for calculations of chlorophyll concentration.

### Physiological parameters

An infrared gas analyzer (IRGA) (CI-340, CID Bio-Science Inc., Camas, WA, USA) was used to measure the net photosynthetic rate (*P*_N_), stomatal conductance (*g*_s_), transpiration rate (*E*). Briefly, the third and fourth leaf from the top of each plant were selected for the measurment. All measurements were taken at PPFD of 1300 μmol m^− 2^ s^− 1^.

### Sugar and protein content

Anthron method was used to determine the sugar content in control and NP-treated leaves [27, 28]. Briefly, 100 mg of control and NP-treated leaf samples was ground in liquid nitrogen. Then, the powder was extracted in 5 cm^3^ of 80% (v/v) ethanol, and centrifuged at 1500 rpm for 10 min (a low speed centrifuge TD6, Pingfan Instruments, Changsha, China). A water bath at 80 °C was used to evaporate the supernatant. Then, 1 cm^3^ of distilled water was added and after 3 min, 4 cm^3^ of a freshly prepared 0.2% (m/v) anthron solution [0.2 g of anthron dissolved in 100 cm^3^ of chilled 75% (m/m) H_2_SO_4_] was added and stirred well by a vortex. The mixture was heated in a water bath at 95 °C for 15 min and then rapidly cooled. The absorbance of the solution was measured at 630 nm using a spectrophotometer (UV-M51 UV-VIS, BEL, BEL Engineering, Monza, Italy). Distilled water was used as blank, and as reference, D-glucose solutions were used for a calibration curve.

On the other hand, Bradford method was used to estimate the protein content in control and NP-treated leaves [29]. For controls and all NP-treated plants, 1 gram of ground leaf tissue sample (ground in liquid nitrogen) was placed in a tube. Then, 3 ml of phosphate buffered saline (PBS) (PH 7.4) was added to each tube, and the tubes were incubated overnight at 4 °C. Then, all tubes were centrifuged (5000 rpm (1118 g), 10 min, 4 °C), and from each tube a 20 μl aliquot of the supernatant was transferred to a 2 ml Eppendorf tube. Then, 980 μl of PBS and 1 ml of Coomassie Brilliant Blue G-250 were added. The absorbance of the solution was measured at 595 nm using a spectrophotometer (UV-M51 UV-VIS, BEL, BEL Engineering, Monza, Italy). The concentrations of protein for the control and NP- treated plants were determined by plotting a standard curve using bovine serum albumin (BSA) as a standard.

### Light and transmission electron microscopy

Mature root and leaf samples (0.5–1.0 cm) from control and NP -treated plants were prepared for light and transmission microscopy following the same protocol as described by Alkhatib et al. [30]. Briefly, samples were fixed in 2.5% (m/v) glutaraldehyde and post-fixed in osmium tetroxide (EMS, Hatfield, PA, USA). After fixing, the samples were dehydrated in an ascending ethanol series (50, 70, 80, 95, and 100%, v/v) for 10–15 min each, and embedded in a freshly prepared Araldite resin. Light microscopy samples were examined using a light microscope (Micros MCX50, Austria) equipped with a digital camera (Amscope, MU100, China). For transmission electron microscopy, ultrathin sections (70 nm) of root and leaf samples were cut with a diamond knife (Diatome Ltd., Bienne, Switzerland) using a Leica ultramicrotome (Leica, Switzerland), stained with uranyl acetate [31] then with lead citrate [32], and examined using a transmission electron microscope (TEM) running at 80 KV (FEI Morgagni 268, FEI, Netherland) equipped with a Mega View III soft imaging system.

### Statistical analysis

Statistical analyses for all parameters (shoot height, root length, leaf area, chlorophyll content, net photosynthetic rate, stomatal conductance, transpiration rate, sugar content, and protein content) were conducted using PC SAS (v. 9.2; SAS Institute, Cary, NC, USA). One-way and two-way ANOVA were used to assess significant differences in sizes, concentrations, and interaction between size and concentration for each parameter tested at α = 0.05. A post hoc Tukey test was used to estimate pairwise comparisons for significant results from the 2-way ANOVA. Graphs of physiological parameters were made using Graph pad Prism 5.

## Additional file

Additional file 1: **Figure S1.** NPs sizes in nm. The sizes of NPs used in this study were measured using ImageJ software to confirm the entry of these NPs inside the plants. (PDF 671 kb)

## Data Availability

The datasets used and/or analysed during the current study are available from the corresponding author on reasonable request.

## Abbreviations

ANOVA: Analysis of varience
E: Transpiration rate
g_s_: stomatal conductance
IRGA: Infrared gas analyzer
NPs: Nanoparticles
P_N_: Net photosynthetic rate
PPFD: Photosynthetic photon flux density
RH: Relative humidity
ROS: Reactive oxygen species
TEM: Transmission electron microscope
