# Supplementary material for: Physio-biochemical and ultrastructural impact of (Fe3O4) nanoparticles on tobacco
Source: BMC Plant Biol. 2019 Jun 13;19:253. doi: 10.1186/s12870-019-1864-1 (PMC6567911; doi:10.1186/s12870-019-1864-1)
Supplement: Supplementary file 1 — Figure S1. NPs sizes in nm. The sizes of NPs used in this study were measured using ImageJ software to confirm the entry of these NPs inside the plants. (PDF 671 kb) [file 12870_2019_1864_MOESM1_ESM.pdf]

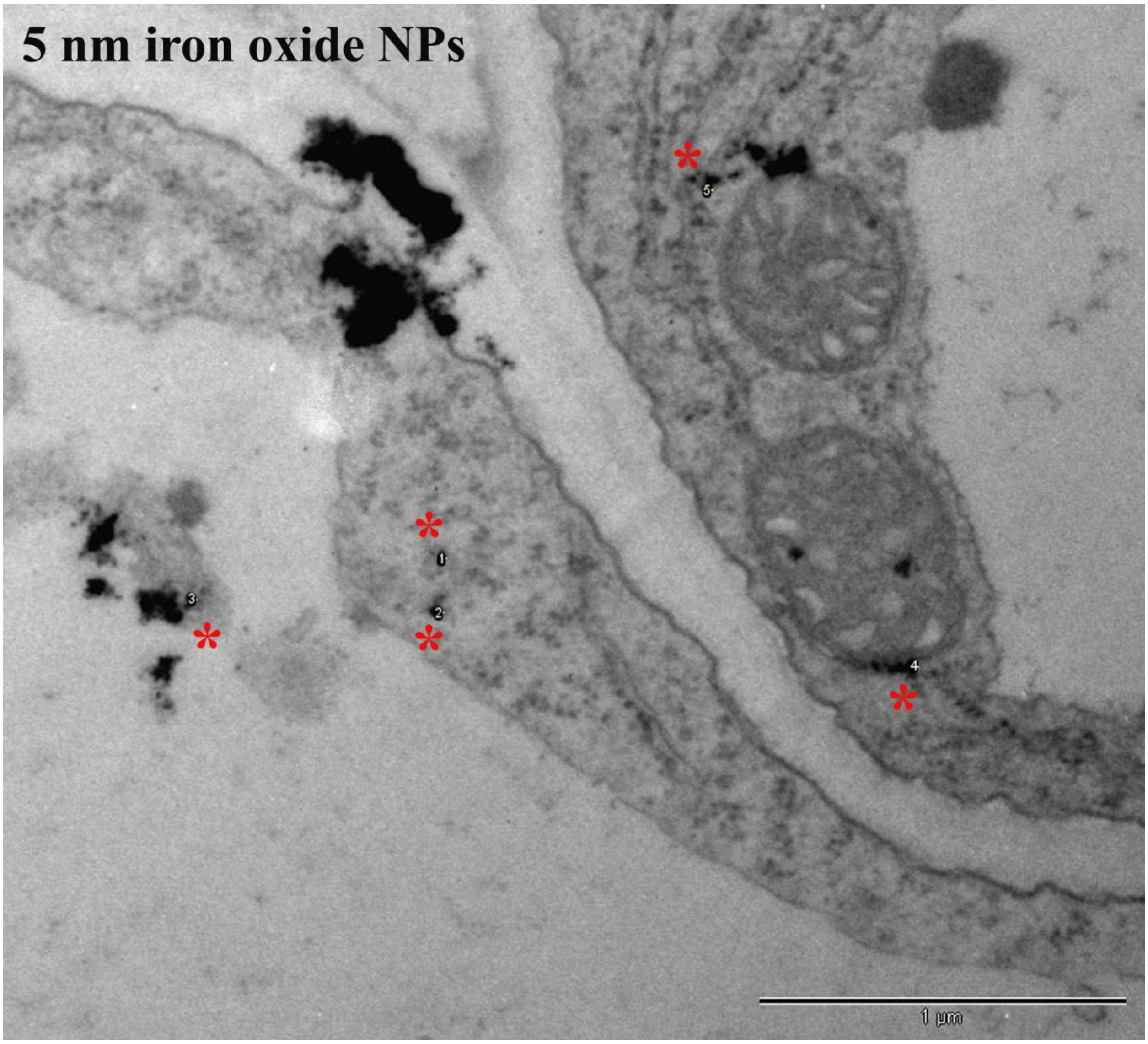

	Label	Area	Angle	Length (nm)
1	thin root; 5;30.jpg	6	180	5
2	thin root; 5;30.jpg	7	180	6
3	thin root; 5;30.jpg	6	168.69	5.099
4	thin root; 5;30.jpg	7	170.538	6.083
5	thin root; 5;30.jpg	8	180	7

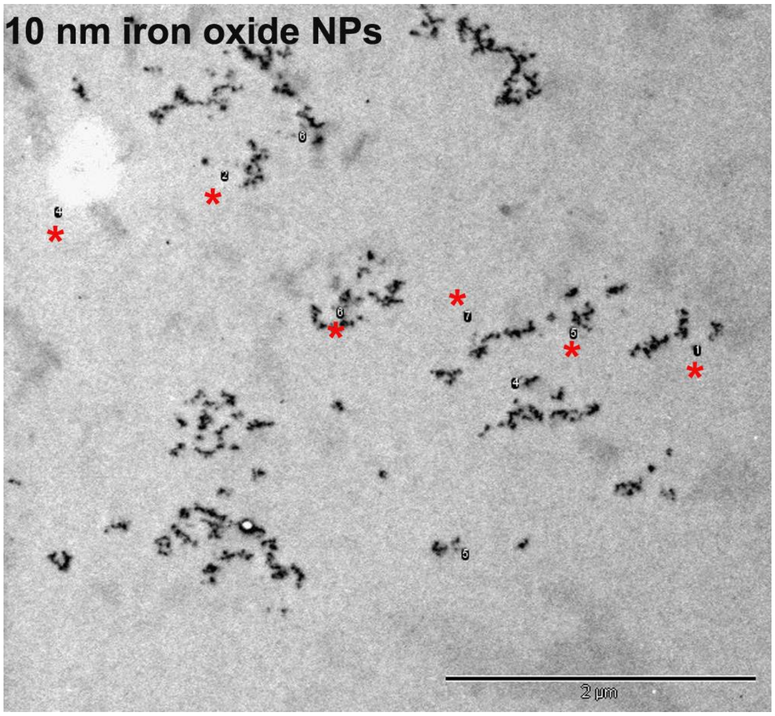

	Label	Area	Angle	Length (nm)
1	10 nm 3 mg/L	186	180	15.748
2	10 nm 3 mg/L	124	135	11.136
3	10 nm 3 mg/L	186	180	15.748
4	10 nm 3 mg/L	186	180	15.748
5	10 nm 3 mg/L	186	180	15.748
6	10 nm 3 mg/L	124	180	7.874
7	10 nm 3 mg/L	186	180	15.748

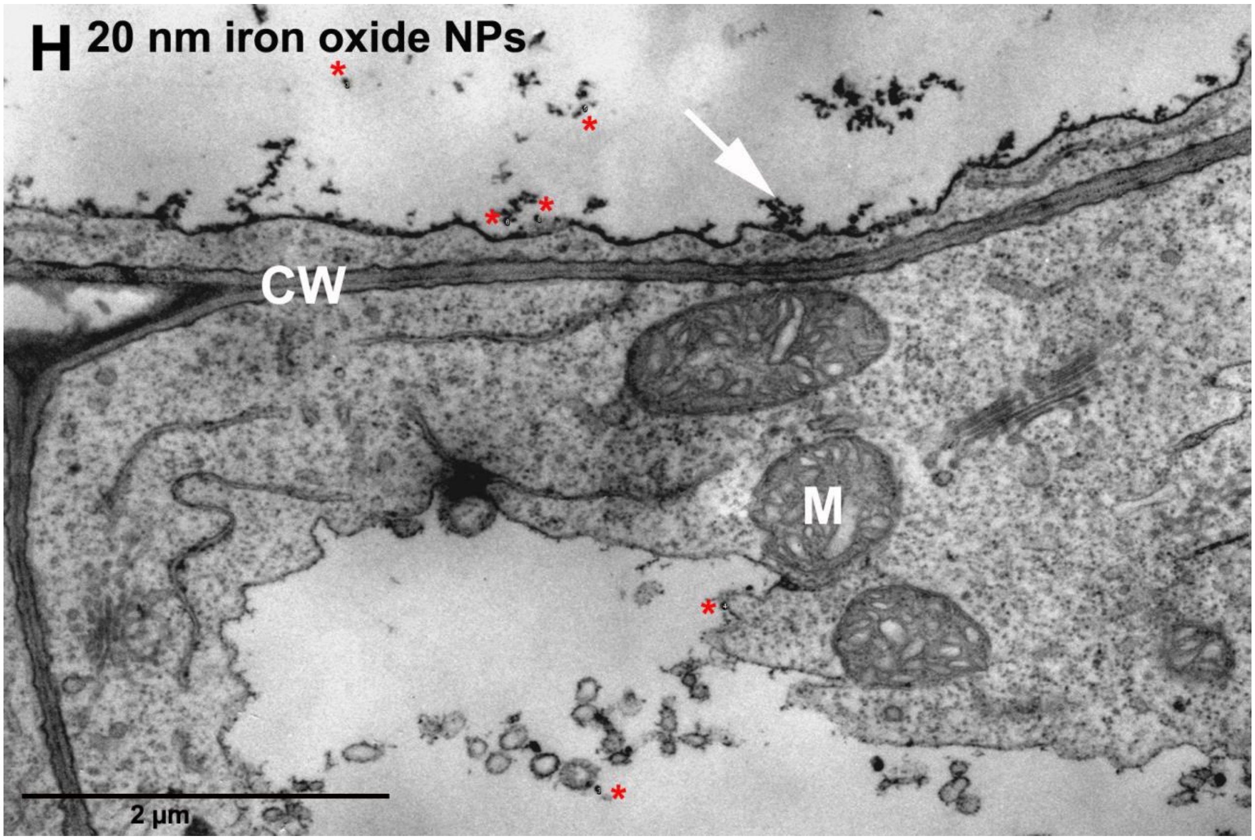

	Label	Area	Angle	Length (nm)
1	thin root;2	102.758	180	22.989
2	thin root;2	102.758	180	22.989
3	thin root;2	132.118	180	30.651
4	thin root;2	117.438	180	25.543
5	thin root;2	102.758	180	22.989
6	thin root;2	102.758	180	22.989

Fig. 2.Supplement: ImageJ measurements for different NPs sizes used in this study (5, 10, 20nm iron oxide)..
